# Effect of repeated bout effect induced by eccentric exercises on joint stiffness endurance in males

**DOI:** 10.14814/phy2.70500

**Published:** 2025-08-09

**Authors:** Soushi Mino, Nobuhiro Yoshizawa, Takanori Teshima, Keitaro Kubo

**Affiliations:** ^1^ Department of Life Science The University of Tokyo Meguro Tokyo Japan; ^2^ Sports Medical Department Nihon Kogakuin College of Hachioji Hachioji Tokyo Japan

**Keywords:** drop jump, fascicle, medial gastrocnemius muscles, ultrasonography

## Abstract

The purpose of this study was to investigate the effect of the repeated bout effect induced by eccentric exercises on joint stiffness endurance. Twelve males performed fatigue tasks (5 sets of 50 hopping) three times: first fatigue task on one side (FFT), second fatigue task on the same side 2 weeks after FFT (SFT), and opposite fatigue task on the contralateral side (OFT). Ankle joint stiffness and electromyographic activities of the plantar flexor and tibial anterior muscles during drop jump using only the ankle joint were measured before and after the fatigue task. Active muscle stiffness was calculated according to changes in estimated muscle force and fascicle length during fast stretching after submaximal isometric contractions. After FFT and OFT, joint stiffness and active muscle stiffness significantly decreased, whereas the electromyographic activities of the plantar flexor and tibial anterior muscles during pre‐landing, eccentric, and concentric phases did not change. After SFT, joint stiffness decreased slightly, whereas active muscle stiffness did not. Furthermore, the relative changes in joint stiffness and active muscle stiffness were significantly lower in SFT than in FFT and OFT. These results suggest that joint stiffness and active muscle stiffness endurance are significantly enhanced 2 weeks after repeated eccentric exercises produced muscle damage.

## 1 INTRODUCTION

Joint stiffness (ratio of joint torque change to joint angle change) is known to be related to the performance during stretch‐shortening cycle (SSC) exercises such as running and jumping (Kuitunen, Komi, & Kyrolainen, 2002; Maloney & Fletcher, 2021; Struzik et al., 2021). Furthermore, in some sports disciplines, the ability to maintain joint stiffness during prolonged SSC exercise (i.e., joint stiffness endurance) is considered an important determinant of competitive performance. For example, if joint stiffness in the lower limb muscles decreases with fatigue during a long‐distance run, then running performance will also decrease, as joint stiffness is related to SSC performance (Kuitunen, Komi, & Kyrolainen, 2002; Maloney & Fletcher, 2021; Struzik et al., 2021). A quarter century ago, Dr. Komi's group reported numerous studies on joint stiffness endurance (Avela et al., 1999; Horira et al., 1996; Horita et al., 1999; Kuitunen, Avela, et al., 2002). According to their series of studies, joint stiffness was significantly reduced after repetitive SSC exercises, and the reduced joint stiffness was associated with decreased electromyographic activities and stretch reflex (especially short latency reflex) of the primary muscles. Horira et al. (1996) reported that a positive correlation was found between the relative changes in the short latency electromyographic activity and the amount of knee joint stiffness during the early post‐landing phase during drop jump.

Previously, we examined the changes in joint stiffness, tendon stiffness, and muscle stiffness under passive and active conditions, as well as electromyographic activities after repeated hopping exercises (Kubo & Ikebukuro, 2019). The results revealed that the decrease in joint stiffness with repeated hopping exercises was related to the reduction of active muscle stiffness but not the changes in tendon properties and electromyographic activities. To date, the determinants of active muscle stiffness endurance are unknown. Meanwhile, Hayes et al. (2004) demonstrated that long‐distance runners with higher muscular endurance in the hip extensor and flexor muscles, as well as knee flexor muscles, exhibited less change in hip angle and stride length (related to the range of motion of the lower limb joints) during a run to exhaustion. Small changes in the range of motion of the lower limb joints during running may indicate less reduction in joint stiffness. Based on the findings of this study (Hayes et al., 2004), we recently determined the relationship between joint stiffness endurance and muscular endurance (assessed by the maximum number of repetitions under low‐load conditions) and failed to find a significant association between the two (Mino, Kosaka, et al., 2025).

In this recent study (Mino, Kosaka, et al., 2025), the group of participants who first measured muscular endurance (and all experienced delayed onset muscle soreness) tended to indicate higher joint stiffness endurance than the group of participants who first measured joint stiffness endurance (and measured muscular endurance later). The two groups had no significant difference in joint stiffness endurance, but the effect size was moderate (d = 0.532). When the same eccentric exercise is performed within several weeks or months, the degree of muscle damage (e.g., muscle soreness, decline in maximal muscle strength) is dramatically reduced (Hyldahl et al., 2017; McHugh, 2003; Nosaka et al., 2001). This protective adaptation is referred to as the repeated bout effect (e.g., Nosaka & Clarkson, 1995). Although the mechanism of the repeated bout effect has yet to be fully clarified, several studies demonstrated that the elongation of fascicle and muscle during the subsequent eccentric exercises was shorter (i.e., lower muscle damage) than that during the first bout (Ho et al., 2021; Lau et al., 2015; Penailillo et al., 2015). In our recent study cited above (Mino, Kosaka, et al., 2025), we noted that the protective adaptation (i.e., repeated bout effect) acquired by eccentric contractions during muscular endurance measurements may have enhanced subsequently measured joint stiffness endurance. However, to obtain confirmation of our speculation, it is necessary to compare joint stiffness endurance between conditions that experienced prior eccentric exercise‐induced muscle damage and those that did not.

In the present study, we aimed to investigate the effect of the repeated bout effect induced by eccentric exercises on joint stiffness endurance. We hypothesized that prior experience with muscle damage from repeated eccentric exercises would enhance joint stiffness endurance.

## 2 METHODS

### 2.1 Participants

The sample size was estimated using the data from our previous studies (Kubo & Ikebukuro, 2019; Mino, Kosaka, et al., 2025), in which the effect of repeated SSC exercises on joint stiffness was determined. Based on an α level of 0.05 and a power (1 – 𝛽) of 0.8, it was shown that at least 11 subjects were necessary for this study. Twelve healthy males (age: 23.3 ± 3.0 years, height: 174.5 ± 5.1 cm, body mass: 67.5 ± 10.6 kg, mean ± SD) voluntarily participated in the present study. Exclusion criteria included a history of injuries and/or surgery on the lower leg muscles and the Achilles tendon. They had not performed any strength training for the plantar flexor muscles (e.g., calf raises) or running (which contributes more to the plantar flexor muscles) in the 6 months before the experiment. They were instructed to refrain from these exercises and to be more careful about injuries to the lower limb muscles during the experiment period. In addition, they were asked to refrain from stretching, massage, or using anti‐inflammatory drugs (e.g., poultices) on the plantar flexor muscles. Before the tests started, written informed consent was acquired. This study was approved by The University of Tokyo's Ethics Committee for Human Experiments, Department of Life Science (Sports Sciences).

### 2.2 Experimental design

**FIGURE 1:**
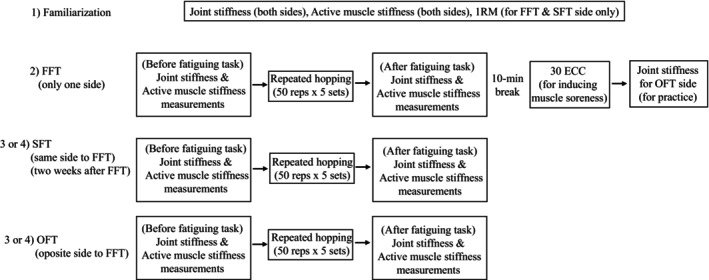
The experimental design of the present study. 1RM, one repetition maximum; ECC, eccentric exercises; FFT, fast fatiguing task; OFT, opposite fatiguing task; SFT, second fatiguing task.

Participants were requested to visit the laboratory on 4 separate days (Figure 1).

#### 2.2.1 Familiarization

Participants were familiarized with the laboratory equipment and protocols used in this study. In addition, they exhibited 2–3 times isometric maximal voluntary contraction (MVC) for plantar flexor muscles, and the highest MVC value was used to determine the target torque values (50% of MVC) during the measurement of active muscle stiffness. Furthermore, the unilateral one‐repetition maximum (1RM) of the plantar flexor muscles was measured for only “first fatiguing task” side (see below). Participants performed 2 sets of 5 repetitions at approximately 50 and 70% of their estimated 1RM with a 2 min rest between sets. Thereafter, the load was progressively increased with a 2 min rest between sets until they were unable to lift it over the range of motion from the fully dorsi‐flexed position to the fully plantar‐flexed position.

#### 2.2.2 First fatiguing task (FFT)

Joint stiffness and active muscle stiffness were measured before and after a fatiguing task for one side only. The right and left sides were randomly assigned (*n* = 6 for right, n = 6 for left).

#### 2.2.3 Second fatiguing task (SFT)

Two weeks after FFT, joint stiffness and active muscle stiffness were measured before and after the fatiguing task on the same side as FFT.

#### 2.2.4 Opposite fatiguing task (OFT)

Ten to 18 days (15.2 ± 2.6 days on average) after FFT, joint stiffness and active muscle stiffness were measured before and after the fatiguing task on the contralateral side to FFT. The interval between FFT and SFT was set at 2 weeks, based on the findings of the previous studies on the effects of the repeated bout effect on lower limb muscles (Chalchat et al., 2022; Connolly et al., 2002; Penailillo et al., 2015). The order of SFT and OFT was random for each participant (SFT first for 10 participants and OFT first for two participants). The days between FFT and SFT were set at exactly 2 weeks for all participants. The days between SFT and OFT were not precisely defined, and the order of SFT and OFT could not be evenly distributed because each participant's convenience was given priority.

In our previous studies (Kubo & Ikebukuro, 2019; Mino, Kosaka, et al., 2025), the fatiguing task employed in this study (repeated unilateral hopping exercises; see below) failed to induce sufficient delayed onset muscle soreness for most participants (unpublished data). Based on this result, to ensure that muscle soreness was induced, 30 plantar flexion eccentric exercises were performed after the measurement with the FFT was completed (see detailed below). In addition, to familiarize participants with OFT measurements, joint stiffness measurements of the contralateral leg (about five times) were performed after FFT measurements and repeated eccentric contractions (see above) were completed.

### 2.3 Fatiguing task

**FIGURE 2:**
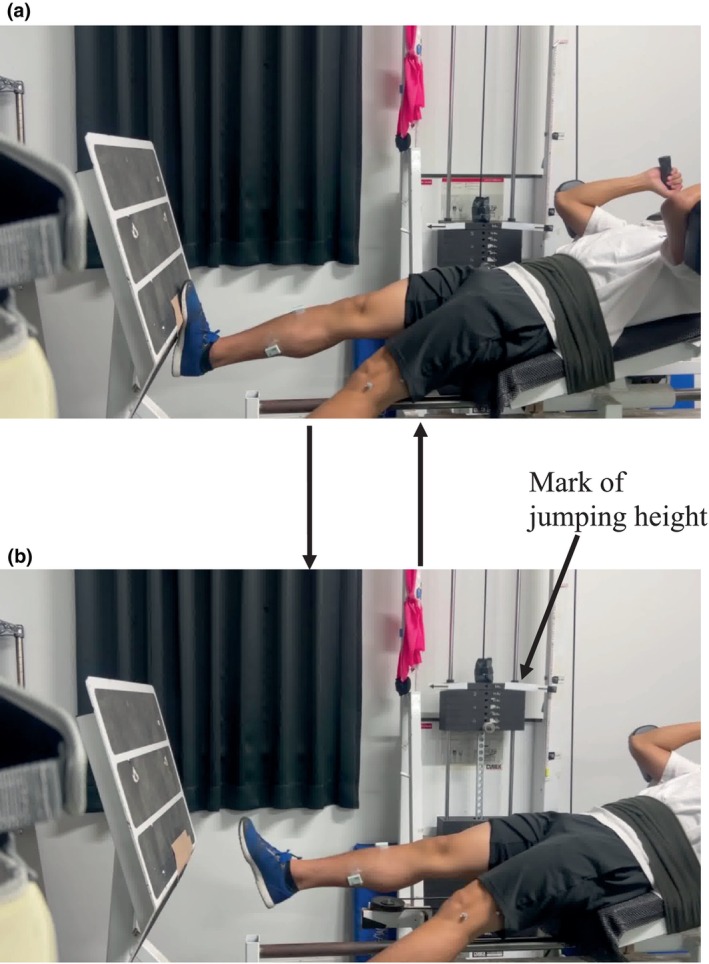
The experimental setup of the fatiguing task (repeated hopping). We obtained permission from this participant to publish the photograph in Figure 2.

Participants performed repeated unilateral hopping exercises using only the ankle joint on a horizontal leg‐press machine (VR‐4100, Cybex Corp., USA), as described in our previous studies (Kubo & Ikebukuro, 2019; Mino, Kosaka, et al., 2025). For each participant, 50% of their body mass was used as the load. At first, they remained in the most plantar flexed position. Then, they exerted plantar flexion torque to the maximal dorsiflexed position and immediately rebounded to start plantar flexion until the toe rose off the footplate of this apparatus. These movements were continuously performed. The fatiguing task consisted of 5 sets of 50 hopping exercises with a 1 min rest period. They were requested to jump as high as possible. During hopping exercises, a smartphone (iPhone 14, Apple, USA) was used to capture images at 60 Hz from the participant's left side (Figure 2). From the images obtained, the jumping height (evaluated by the distance traveled by the load plate connected to the sledge apparatus's seat from a position at 90 deg. of ankle joint angle) was measured using open‐source image analysis software (ImageJ, NIH, Bethesda, MD, USA).

For FFT, a 10‐min break was taken after the end of the measurements after the fatiguing task (repeated hopping). Participants then performed 30 plantar flexion eccentric exercises (10 repetitions × 3 sets with 2–3 min rest between sets) at 100% of 1RM in order to induce delayed onset muscle soreness, as mentioned in the previous section. Participants were instructed to move their ankle joint from the fully plantar flexed position to the fully dorsiflexed position at a constant velocity of around 3 s. An investigator raised the leg press machine's backrest as they reverted to the initial position. Muscle soreness of the plantar flexor muscles (subjective symptoms of muscle pain in daily life) was assessed using a visual analog scale of a 100 mm line that represented “no pain” at 0 mm and “very painful” at 100 mm (Connolly et al., 2002; Ho et al., 2021). Muscle soreness was recorded over 10 days for FFT and 5 days for SFT and OFT.

### 2.4 Joint stiffness during drop jump

Participants performed a unilateral drop jump using only the ankle joint on the custom‐designed sledge apparatus (AO‐3000K, Applied Office, Japan), as described in our previous studies (Kosaka et al., 2023; Mino, Kosaka, et al., 2025). The vertical component of the ground reaction force (Fz) was recorded from the force plate (Kistler, 9281B, Switzerland) attached to the footplate of the sledge apparatus. The ground reaction force signals were A/D converted at a 1 kHz sampling rate (PowerLab, AD Instrument). Retroreflective markers were attached to the trochanter major, lateral malleolus, knee center of rotation, and fifth metatarsophalangeal joint. During jumping, they were filmed from the right (using the right ankle) or left (using the left ankle) sides in the sagittal plane with a digital high‐speed video camera (HAS‐U1, DITECT, Tokyo, Japan) at a sampling frequency of 200 Hz.

**FIGURE 3:**
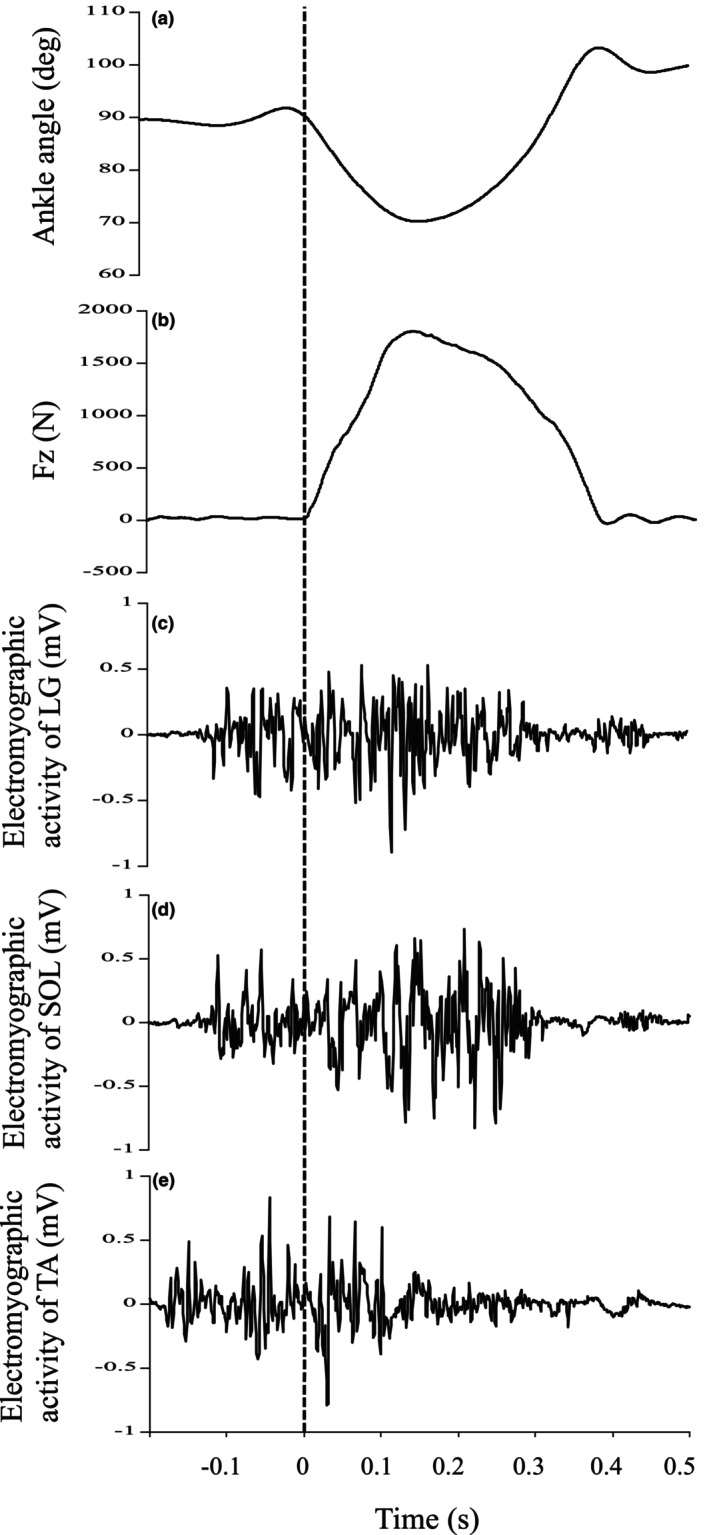
Typical example of the ankle angle (a), vertical reaction force (Fz) (b), and electromyographic activities of lateral gastrocnemius muscle (LG) (c), soleus muscle (SOL) (d), and tibial anterior muscle (TA) (e) during the measurement of joint stiffness (drop jump). The vertical dotted line indicates landing.

Before the test, participants had enough practice (submaximal jumps) to get used to the test procedure. With at least 1 min between trials, the test was administered five times before the fatigue task and three times after the fatigue task. With the aid of the device, the sliding table was raised 20 cm from the force plate's surface to the sole of their foot. They were dropped from a 20 cm height. After landing on the force plate's edge, they used eccentric plantar flexion to stop their downward motion. Then, they took off with concentric plantar flexion. A typical example of the raw data (ankle angle, vertical reaction force, and electromyographic activities of the measured muscles) is shown in Figure 3. We excluded trials with knee joint flexion (>5 deg) based on images taken by the high‐speed video camera, as described in our previous study (Kubo & Ikebukuro, 2019).

The ankle joint angle was measured using open‐source image analysis software (ImageJ, NIH, Bethesda, MD, USA). The ankle's range of motion (ROM) from touchdown to the lowest position was determined. The following formula was used to determine the ankle joint torque (TQ) during the drop jump (Kawakami et al., 2002; Kubo et al., 2000):

$$\mathrm{TQ}=\mathrm{Fz}\cdot L\cdot \cos\;\left(\mathrm{AJ}\right)$$

where Fz, L, and AJ denote the vertical component of the ground reaction force, the length from the medial malleolus to the ball of the foot, and the ankle joint angle. Ankle joint stiffness was calculated as the change in joint torque divided by the change in the ankle joint angle during the eccentric phase (Kosaka et al., 2023; Kubo & Ikebukuro, 2019; Mino, Kosaka, et al., 2025). The measured values were the mean of two trials with the highest and second‐highest joint stiffness. Unfortunately, due to the loss of one participant's high‐speed video camera image, only 11 participants were available for the variables of joint stiffness measurement. In our previous study (Kubo & Ikebukuro, 2019), the repeatability of measurement of joint stiffness was investigated on 2 separate days involving 11 males. The intraclass correlation coefficient (95% confidence interval (CI)) and coefficient of variation were 0.874 (95% CI 0.784–0.968) and 10.5%, respectively.

During the drop jump, electromyographic activity (EMG) was recorded at a 1 kHz sampling rate utilizing a wireless EMG monitoring device (BioLog DL‐5500, S&ME, Japan). Surface electrodes (DL‐510, S&ME, Japan) were attached to the skin over the muscle belly of the lateral gastrocnemius muscle (LG), soleus muscle (SOL), and tibial anterior muscle (TA) using special double‐sided tape. The midbelly of each muscle was confirmed using B‐mode ultrasonography. Because the ultrasound probe was attached over the belly of the medial gastrocnemius muscle (MG) during the measurement of active muscle stiffness (see below), the EMG of the MG was not measured. The raw data were band‐pass filtered between 20 and 500 Hz. EMG amplitude was rectified and averaged for pre‐landing (100 ms preceding ground contact; e.g., Horira et al., 1996), eccentric, and concentric phases based on the ankle joint angle (mEMG). In addition, the mean mEMG in LG and SOL was defined as the mEMG of the plantar flexor muscles (PF).

### 2.5 Active muscle stiffness

**FIGURE 4:**
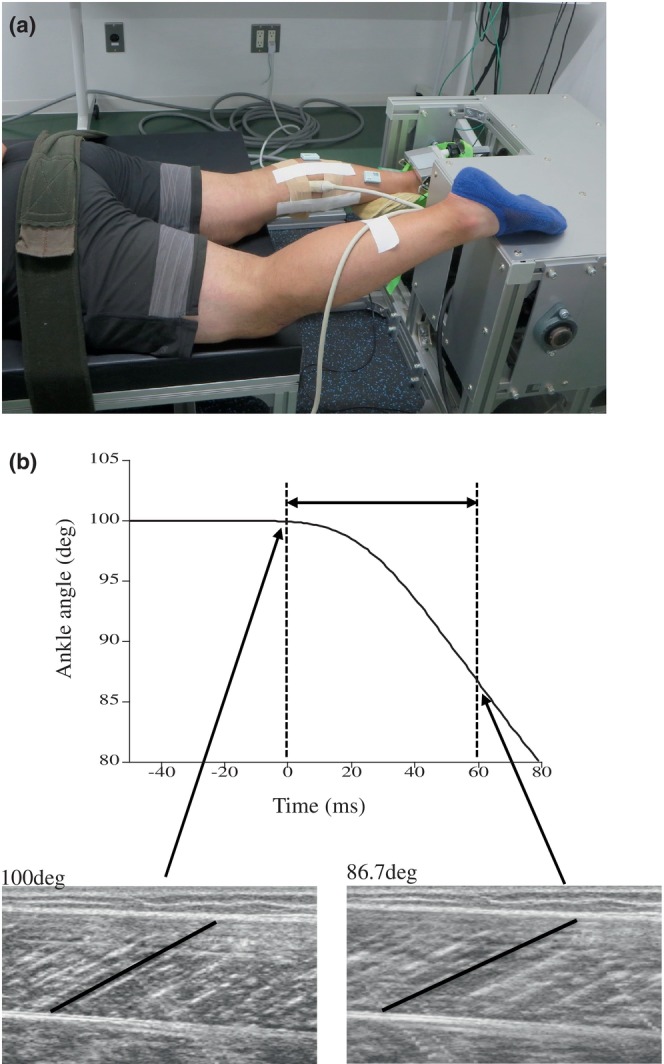
(a) The experimental setup of the measurement of active muscle stiffness. We obtained permission from this participant to publish the photograph in (a). (b) Typical example of change in ankle angle and longitudinal ultrasonic images of the medial gastrocnemius muscle during the measurement of active muscle stiffness.

A specially designed dynamometer (T.K.K.S‐18035, Takei Scientific Instruments Co., Ltd., Niigata, Japan; Figure 4a) was used to measure active muscle stiffness using the procedure described in our previous studies (Kosaka et al., 2023; Kubo et al., 2020; Mino, Tanaka, et al., 2025). Participants were lying prone on a medical bed, with their foot tightly fastened to a footplate of the dynamometer using two straps. The ankle joint was set at 100 deg. (anatomically neutral positions are defined as 90 deg.; angles greater than 90 deg. indicate plantar flexion) with the knee joint at full extension. They performed the measurements of active muscle stiffness at 300 deg. s^−1^. The reason for setting angular velocity was to get closer to the angular velocity during the joint stiffness measurement (i.e., drop jump). The dynamometer was set up to apply dorsiflexion from 100 to 80 deg. Before the test, participants practiced one or two times for the measurements. Three measurements of active muscle stiffness at 50% of MVC were made. Throughout fast dorsiflexion, they were instructed to maintain the activation level until the movement was completed. Periods of 60 ms after the onset of stretch were analyzed, and the range of motion during this period was approximately 13.3 deg. (e.g., Kubo et al., 2020). In addition, two further measurements were conducted at 0% MVC (relaxed condition) for data correction purposes. The averaged torque during the relaxed condition (caused by inertia and passive elasticity) was subtracted from the averaged torque at 50% MVC (e.g., Kubo, 2014). As previously mentioned (e.g., Kubo, 2014), muscle force was calculated from the torque that the dynamometer had measured. When measuring the active muscle stiffness, the fascicle length of the MG was assessed employing ultrasonic equipment (Prosound α7, Hitachi Aloka Medical, Tokyo, Japan) (Figure 4b). The apparatus's computer memory contained ultrasonic images stored at 100 Hz. The ultrasonic images were overlapped with an electric signal to synchronize them to the torque and joint angle. Active muscle stiffness was determined by analyzing the slope of muscle force–fascicle length during the examined period (e.g., Kubo, 2014). A representative value was the average of three measurements. In our previous study (Kubo et al., 2020), the repeatability of measurement of active muscle stiffness was investigated on 2 separate days involving eight males. The intraclass correlation coefficient (95% CI) and coefficient of variation were 0.880 (95% CI 0.738–0.981) and 11.2%, respectively.

### 2.6 Statistical analysis

The means ± standard deviation are used to report descriptive data. Regarding the mean jumping heights during the fatigue tasks and relative changes in the measured variables before and after the fatigue tasks, a one‐way analysis of variance (ANOVA) with repeated measures was used to detect significant differences among FFT, SFT, and OFT. Regarding the other variables, a two‐way ANOVA with repeated measures (3 [conditions] × 2 [times]) was used to analyze data. The F ratios for main effects and interactions were considered significant with *p* < 0.05. Significant differences among means with *p* < 0.05 were detected using Bonferroni's post hoc test. The homogeneity of variance in an ANOVA was assessed using Mauchly's sphericity test. The Greenhouse–Geisser correction was applied in situations when the sphericity assumption was violated. The effect size was determined using partial eta‐squared (pη^2^). For every test, the significance level was set with *p* < 0.05. IBM SPSS Statistics (version 27) was used for statistical calculations.

## 3 RESULTS

**FIGURE 5:**
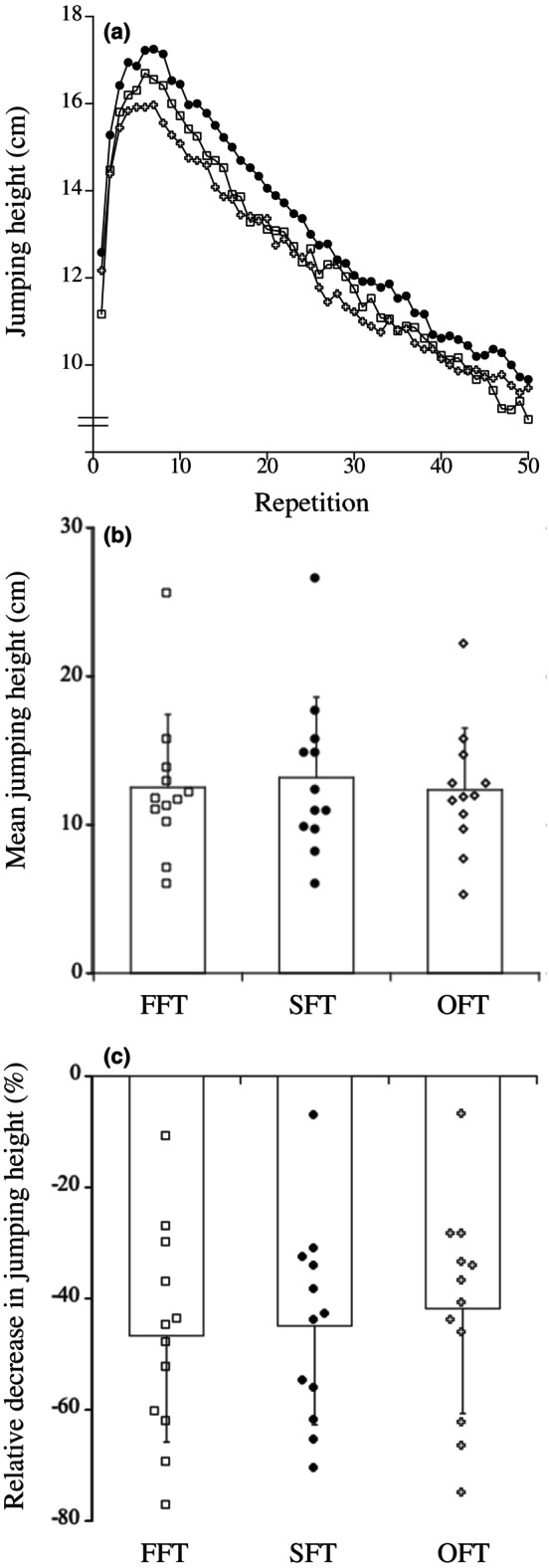
(a) Changes in jumping height during fatiguing tasks (open square; FFT, closed circle; SFT, open cross; OFT). Standard deviation bars did not represent clarity. (b) Mean jumping heights for FFT, SFT, and OFT. C: Relative decrease in jumping height for FFT, SFT, and OFT. FFT, fast fatiguing task; OFT, opposite fatiguing task; SFT, second fatiguing task.

**FIGURE 6:**
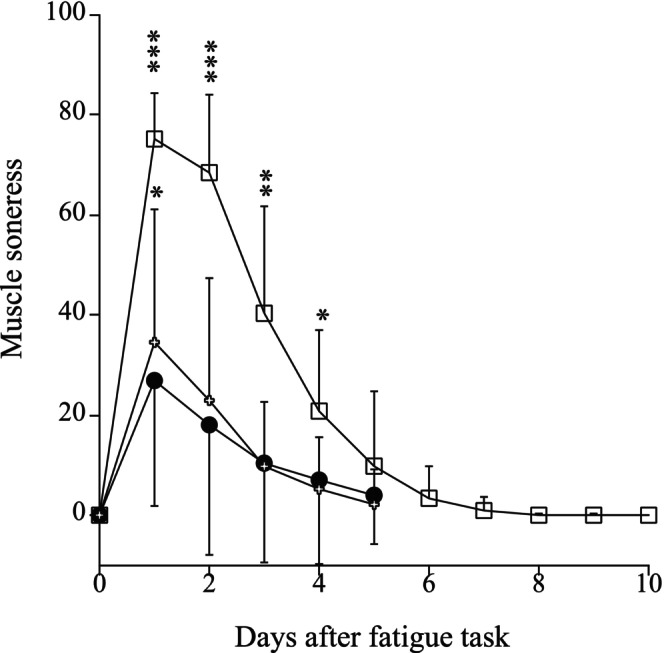
Changes in muscle soreness before and after fatiguing tasks (open square; FFT, closed circle; SFT, open cross; OFT). FFT, fast fatiguing task; OFT; opposite fatiguing task; SFT, second fatiguing task. Significant difference from before fatiguing task (0 day) (*p < 0.05, **p < 0.01, ***p < 0.001).

The changes in jumping height during fatiguing tasks, expressed as absolute values for the mean of five sets, are shown in Figure 5a. There were no differences in the mean jumping height (*p* = 0.432, pη^2^ = 0.074) and relative decrease in jumping height (mean of five sets) (*p* = 0.237, pη^2^ = 0.123) during fatiguing tasks among FFT, SFT, and OFT (Figure 5b,c). For FFT, muscle soreness peaked 1–2 days after the fatiguing task, then recovered to the pre‐fatiguing task value after 5 days, whereas that increased only after 1 day of the fatiguing task (*p* = 0.051 for SFT, *p* = 0.013 for OFT) and recovered to the pre‐fatiguing task value after 2 days for SFT and OFT (Figure 6).

**FIGURE 7:**
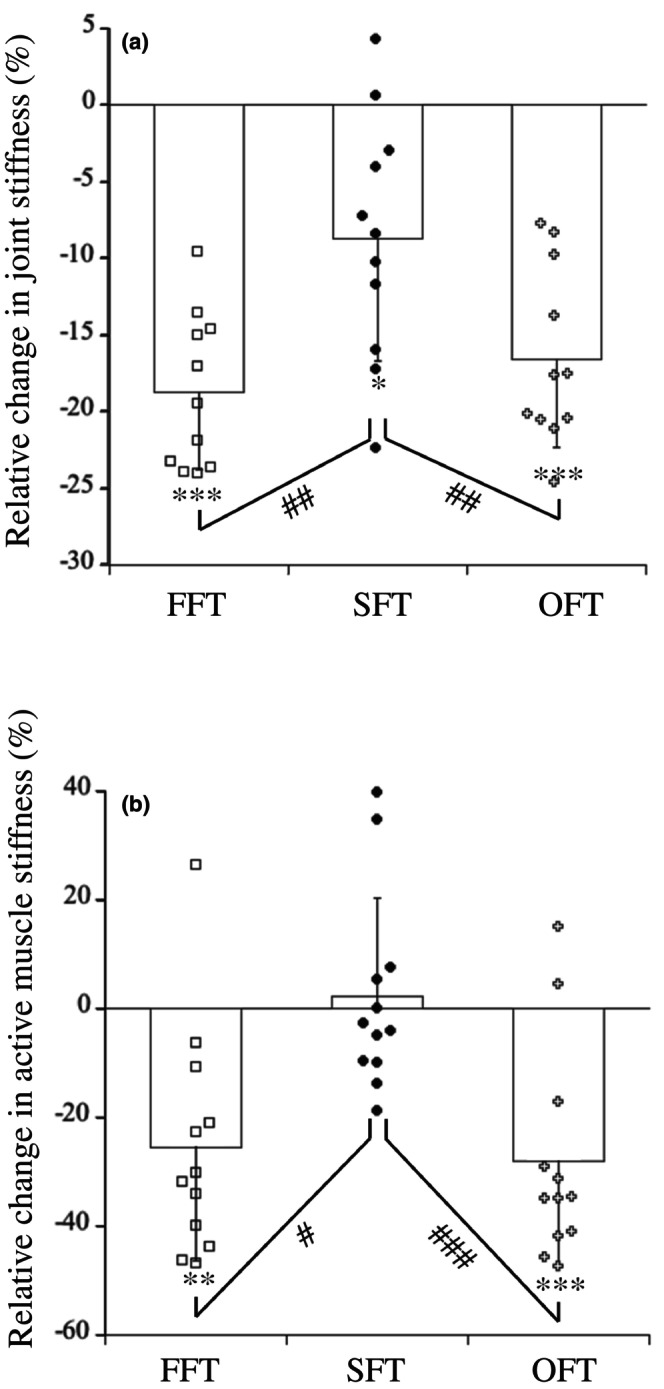
Relative change in joint stiffness (a) and active muscle stiffness (b) after FFT, SFT, and OFT. Significant difference from before fatiguing task (*p < 0.05, **p < 0.01, ***p < 0.001). Significant difference among FFT, SFT, and OFT (#*p* < 0.05, ##*p* < 0.01, ###*p* < 0.001).

Table 1 shows the measured variables during the measurement of joint stiffness before and after fatiguing tasks. The duration of contact significantly increased after all fatiguing tasks. The mEMG values of PF and TA during pre‐landing, eccentric, and concentric phases did not change after all fatiguing tasks. Regarding peak torque and range of motion during the eccentric phase, the effect of time was significant, although the effects of condition and the interaction between condition and time were not. Regarding joint stiffness, the effects of time and the interaction between time and condition were significant, although the effect of condition was not. Both FFT, SFT, and OFT showed a significant reduction in joint stiffness (*p* < 0.001 for FFT, *p* = 0.013 for SFT, *p* < 0.001 for OFT), but the relative change in joint stiffness was significantly lower in SFT (−8.7 ± 8.0%) than in FFT (−18.8 ± 5.1%, *p* = 0.003) and OFT (−16.5 ± 5.8%, *p* = 0.008) (*p* < 0.001, pη^2^ = 0.552; Figure 7a).

**TABLE 1:** Measured variables during the measurement of joint stiffness before and after fatigue tasks. Mean (SD).

	FFT		SFT		OFT		Main effect		
	Before	After	Before	After	Before	After	Condition	Time	Interaction
Duration of contact (ms)	343.6 (20.5)	383.0 (25.3)	348.0 (20.2)	372.7 (32.9)	345.2 (28.9)	376.1 (41.0)	*p* = 0.783, pη^2^ = 0.014	*p* < 0.001, pη^2^ = 0.817	*p* = 0.187, pη^2^ = 0.154
mEMG of PF during prelanding (mV · s^−1^)	0.163 (0.080)	0.152 (0.075)	0.164 (0.064)	0.168 (0.072)	0.224 (0.183)	0.215 (0.161)	*p* = 0.157, pη^2^ = 0.189	*p* = 0.499, pη^2^ = 0.047	*p* = 0.559, pη^2^ = 0.057
mEMG of PF during eccentric phase (mV · s^−1^)	0.307 (0.140)	0.301 (0.179)	0.303 (0.140)	0.336 (0.154)	0.346 (0.183)	0.345 (0.224)	*p* = 0.774, pη^2^ = 0.025	*p* = 0.376, pη^2^ = 0.079	*p* = 0.516, pη^2^ = 0.064
mEMG of PF during concentric phase (mV · s^−1^)	0.269 (0.087)	0.272 (0.097)	0.269 (0.110)	0.269 (0.114)	0.277 (0.153)	0.270 (0.129)	*p* = 0.944, pη^2^ = 0.001	*p* = 0.931, pη^2^ = 0.001	*p* = 0.883, pη^2^ = 0.012
mEMG of TA during prelanding (mV · s^−1^)	0.164 (0.079)	0.157 (0.072)	0.143 (0.048)	0.129 (0.084)	0.123 (0.058)	0.126 (0.074)	*p* = 0.185, pη^2^ = 0.155	*p* = 0.621, pη^2^ = 0.025	*p* = 0.782, pη^2^ = 0.024
mEMG of TA during eccentric phase (mV · s^−1^)	0.131 (0.056)	0.116 (0.047)	0.122 (0.044)	0.113 (0.040)	0.139 (0.074)	0.134 (0.085)	*p* = 0.535, pη^2^ = 0.061	*p* = 0.481, pη^2^ = 0.051	*p* = 0.948, pη^2^ = 0.005
mEMG of TA during concentric phase (mV · s^−1^)	0.202 (0.257)	0.112 (0.042)	0.105 (0.038)	0.096 (0.035)	0.118 (0.043)	0.112 (0.044)	*p* = 0.225, pη^2^ = 0.143	*p* = 0.138, pη^2^ = 0.206	*p* = 0.293, pη^2^ = 0.110
Peak torque during eccentric phase (Nm)	201.6 (42.0)	185.5 (40.9)	196.2 (41.3)	193.7 (45.0)	193.9 (37.6)	187.0 (35.9)	*p* = 0.694, pη^2^ = 0.023	*p* = 0.007, pη^2^ = 0.536	*p* = 0.058, pη^2^ = 0.248
Range of motion during eccentric phase (deg)	29.7 (4.6)	33.7 (4.4)	31.0 (5.0)	33.6 (5.8)	30.5 (6.0)	35.2 (5.6)	*p* = 0.563, pη^2^ = 0.056	*p* < 0.001, pη^2^ = 0.825	*p* = 0.053, pη^2^ = 0.254
Joint stiffness (Nm · deg.^−1^)	7.0 (1.9)	5.7 (1.6) ***	6.6 (1.9)	6.1 (2.2) *	6.6 (1.7)	5.5 (1.4) ***	*p* = 0.645, pη^2^ = 0.043	*p* < 0.001, pη^2^ = 0.886	*p* < 0.001, pη^2^ = 0.497

*Note*: *Significantly different from before (*p < 0.05, ***p < 0.001).

Abbreviations: mEMG, mean electromyographic activities; PF, plantar flexor muscles; TA, tibial anterior muscle.

Table 2 shows the measured variables during the measurement of active muscle stiffness before and after fatiguing tasks. Regarding change in torque, the effects of condition, time, and the interaction between condition and time were not significant. Regarding change in fascicle length and active muscle stiffness, the effect of the interaction between condition and time was significant. Change in fascicle length significantly increased after FFT (*p* = 0.012) and OFT (*p* < 0.001), and active muscle stiffness significantly decreased after FFT (*p* = 0.002) and OFT (*p* < 0.001). On the other hand, change in fascicle length (*p* = 0.885) and active muscle stiffness (*p* = 0.508) did not change after SFT. The relative change in active muscle stiffness was significantly lower in SFT (+2.2 ± 18.1%) than in FFT (−25.4 ± 21.1%, *p* = 0.014) and OFT (−28.0 ± 19.6%, *p* < 0.001) (*p* < 0.001, pη^2^ = 0.564; Figure 7b).

**TABLE 2:** Measured variables during the measurement of active muscle stiffness before and after fatigue tasks. Mean (SD).

	FFT		SFT		OFT		Main effect		
	Before	After	Before	After	Before	After	Condition	Time	Interaction
Increase in torque (Nm)	49.1 (13.3)	45.9 (13.3)	47.2 (13.4)	47.5 (12.2)	52.3 (9.6)	53.0 (7.9)	*p* = 0.132, pη^2^ = 0.186	*p* = 0.532, pη^2^ = 0.037	*p* = 0.098, pη^2^ = 0.190
Increase in fascicle length (mm)	4.5 (1.3)	5.6 (1.0) *	4.2 (1.0)	4.3 (0.9)	3.7 (0.8)	5.4 (1.0) ***	*p* = 0.041, pη^2^ = 0.253	*p* < 0.001, pη^2^ = 0.735	*p* < 0.001, pη^2^ = 0.485
Active muscle stiffness (N · mm^−1^)	46.0 (15.5)	33.0 (10.1) **	47.8 (19.5)	46.6 (15.7)	58.9 (18.0)	40.2 (9.3) ***	*p* = 0.078, pη^2^ = 0.207	*p* < 0.001, pη^2^ = 0.712	*p* < 0.001, pη^2^ = 0.501

*Note*: *Significantly different from before (*p < 0.05, **p < 0.01, ***p < 0.001).

## 4 DISCUSSION

This study examined the influence of repeated bout effect induced by eccentric exercises on joint stiffness endurance. Three fatiguing tasks must be performed similarly to accomplish this research objective. In the present study, there were no differences in the average jumping height and the relative decrease in jumping height among the three fatiguing tasks (FFT, SFT, and OFT) (Figure 5). Therefore, all fatiguing tasks in this study imposed equivalent loads on the target muscle group (plantar flexor muscles).

Similar to our previous study (Kubo & Ikebukuro, 2019), joint stiffness and active muscle stiffness were significantly decreased by repeated hopping exercises for FFT and OFT, although the EMG activities during the three phases (pre‐landing, eccentric, and concentric) did not change. Furthermore, the relative decrease in joint stiffness after repeated hopping exercises was within the range of values (20%–30%) reported in several relevant previous studies (Horira et al., 1996; Horita et al., 1999; Kuitunen, Avela, et al., 2002; Lazaridis et al., 2018). Considering the results of our previous study (Kubo & Ikebukuro, 2019) and this study together, it is entirely fair to say that joint stiffness endurance is primarily due to the changes in active muscle stiffness but not the changes in electromyographic activities of agonist and antagonist muscles (and tendon mechanical properties). In addition, more recently, we reported that active muscle stiffness significantly decreased after repeated eccentric contractions but not after repeated isometric contractions (Mino, Tanaka, et al., 2025). Accordingly, we may say that the repetition of eccentric contractions during SSC exercises reduces joint stiffness and active muscle stiffness.

To our knowledge, this is the first study to observe that joint stiffness (and active muscle stiffness) endurance is significantly enhanced 2 weeks after repeated eccentric exercises produce muscle damage. In the present study, 30 eccentric contractions with 100% 1RM after FFT measurement induced muscle soreness, as reported in many previous studies (Chalchat et al., 2022; Ho et al., 2021; Penailillo et al., 2013) (Figure 6), although other indicators of muscle damage (e.g., muscle strength, creatine kinase) were not measured. Therefore, we may say that the repeated bout effect by eccentric exercise shown in many previous studies (e.g., Nosaka & Clarkson, 1995) has occurred. Previous studies have indicated that the mechanisms of the repeated bout effect may be associated with the adaptations of the neural system, muscle‐tendon complex, and connective tissue composition (extracellular matrix) (e.g., Hyldahl et al., 2017). In addition, several studies reported that the elongation of fascicle and muscle during the subsequent eccentric exercises was shorter than during the first bout (Ho et al., 2021; Lau et al., 2015; Penailillo et al., 2015). It seems reasonable to suppose that these results may be related to the lower muscle damage after the second eccentric exercise reported in many previous studies (Chen et al., 2016; Hyldahl et al., 2017; Pincheira et al., 2018). Unfortunately, we did not measure fascicle length during the fatiguing tasks (repeated hopping) in the present study. On the other hand, the fascicle elongation during active muscle stiffness measurement was less after the fatiguing task of SFT compared to FFT and OFT (Table 2). Therefore, it can be said that the results of this study partially support the results of the previous studies mentioned above (Ho et al., 2021; Lau et al., 2015; Penailillo et al., 2015).

However, other previous studies demonstrated that fascicle dynamics during the second eccentric contractions were not different from those during the first bout (Hoffman et al., 2016; Pincheira et al., 2018). In these studies, the interval between the first and second bouts was very short (1 week) compared to the previous studies mentioned above (2–4 weeks, e.g., Penailillo et al., 2015), and it may not have sufficiently adapted in the muscles after the first bout. Indeed, these studies found a protective effect on muscle soreness but not maximal muscle strength. In addition, Pincheira et al. (2018) suggested that adaptations in non‐contractile elements of the muscle may better explain the protection of the muscle during the repeated bout. Several studies showed that type I and III collagen were elevated after the first eccentric exercise and suggested that remodeling and strengthening of the extracellular matrix played a role in the protective effect during the second eccentric exercise (Hyldahl et al., 2017; Mackey et al., 2011). Unfortunately, the experimental data measured in this study do not allow for any mention of connective tissue (extracellular matrix) adaptation. In any case, the changes in durability (i.e., endurance) of mechanical properties of the extracellular matrix may have contributed to the inhibition of fascicle length increases during repeated SSC exercises of the second bout.

Other mechanisms for the repeated bout effect include the addition of sarcomeres in series, that is, an increase in muscle fiber length (Brockett et al., 2001; Proske & Morgan, 2001). Increased muscle fiber and fascicle lengths are thought to result in less muscle strain (i.e., associated with less muscle damage) during eccentric exercises. Unfortunately, we did not measure fascicle length at rest in this study. However, no significant difference was found between FFT (37.2 ± 6.5 mm) and SFT (38.7 ± 6.7 mm) during the measurement of active muscle stiffness, where the ultrasonic images of MG were acquired with 50% MVC maintained before rapid dorsiflexion (*p* = 0.132, *d* = 0.228 according to the paired t‐test; data not shown). According to the results of previous studies (Hoffman et al., 2016; Penailillo et al., 2015), there was no difference in fascicle length at rest before the first and second eccentric exercises. Therefore, we cannot say that adapting sarcomere number (fascicle length) is a possible mechanism for improving joint stiffness endurance.

Regarding less muscle elongation during the second eccentric exercise, Lau et al. (2015) speculated that the muscle became stiffer and the tendon became more compliant after the initial eccentric exercise. Previous studies using ultrasound elastography reported that muscle stiffness at rest increased immediately after repeated eccentric contractions and over the next few days (Chalchat et al., 2022; Ema et al., 2021; Heales et al., 2017). However, these muscle mechanical properties were obtained under resting conditions, not active conditions. In the present study, there was no significant difference in active muscle stiffness between pre‐fatigue task values at FFT (46.0 ± 15.5 N mm^−1^) and SFT (47.8 ± 19.5 N mm^−1^) (*p* = 0.690, *d* = 0.103 according to the paired t‐test). Regarding the tendon mechanical properties, our previous studies demonstrated that 8 weeks of training were insufficient and 12 weeks were needed to increase tendon stiffness with strength training (Kubo et al., 2010, 2012). Considering these points, it is unlikely that the 2 weeks between FFT and SFT altered the mechanical properties of the muscles and tendons and contributed to improved joint stiffness endurance.

Several studies demonstrated that the EMG activities during the second eccentric exercise were lower than during the first bout (Penailillo et al., 2013, 2015). However, other studies indicated that there were no significant differences in the EMG activities between the initial and subsequent bouts, suggesting that the level of drive to the muscles was similar between the two bouts (McHugh et al., 2001; Muthalib et al., 2011). The present study found no significant differences in mEMG during the three phases (pre‐landing, eccentric, and concentric) between before and after all fatiguing tasks (Table 1). On the other hand, the relative change in mEMG of PF during the eccentric phase of drop jump after fatiguing tasks (no significant change after fatiguing task as mentioned above) tended to be higher for SFT (+13.1%) than for FFT (−5.8%; *p* = 0.054) and OFT (−2.7%; *p* = 0.078) (data not shown). This result suggested that the EMG activity was higher during the eccentric phase of drop jump after fatiguing task for SFT compared to FFT and OFT, which could also be interpreted as more tendon elongation and lower fascicle elongation. To confirm the above inference, future studies should increase the number of cases and examine the dynamics of the muscle‐tendon complex during drop jumps as well.

There were several limitations to the present study. Firstly, all participants were untrained in the present study. Thus, the obtained results in this study may be different for participants with experience of training or competitive athletes. Previous studies showed that the changes in the measured variables of muscle damage (e.g., maximal muscle strength and creatine kinase) due to repeated eccentric exercises differed between upper and lower extremity muscle groups (Chen et al., 2019). The reason for this result was that the lower extremity muscle groups were subjected to more eccentric contractions in daily life than the upper extremity muscle groups. Therefore, the degree of training experience of the participants may have a significant impact on the obtained results, although there are no studies comparing muscle damage associated with eccentric contractions in trained and untrained persons to the best of our knowledge. Furthermore, to obtain knowledge that will contribute to improving sports performance, it is necessary to validate the results with athletes with extensive training experience. Secondly, the results for OFT in this study may be influenced by the repeated effect (protective effect) in the contralateral limb (i.e., FFT and SFT). Several studies demonstrated that the repeated bout effect of eccentric exercises transferred to the contralateral limb (Chen et al., 2016; Howatson & van Someren, 2007; Hyldahl et al., 2017). However, the degree of repeated bout effect of eccentric exercise seen in the contralateral limb was considerably lower than that seen in the ipsilateral side (e.g., Chen et al., 2016), and some results had been reported to rule out a transfer of the repeated bout effect to the contralateral limb (Brown et al., 2023; Connolly et al., 2002). In addition, as mentioned above, when comparing the impact of prior eccentric exercise on joint stiffness endurance in different participant groups, the degree of training experience of each participant will influence the results of this study. Therefore, this is the most reasonable experimental design for achieving the objectives of this study.

In conclusion, the results of this study showed that joint stiffness and active muscle stiffness endurance significantly increased 2 weeks after repeated eccentric contractions produced muscle damage. Based on the results of this study, in some sports disciplines (e.g., long‐distance running and volleyball), where SSC exercises are repeated, experiencing muscle damage from prior eccentric exercises and gaining the repeated bout effect may enhance joint stiffness endurance and improve performance. This point needs to be verified by future longitudinal studies.

## CONFLICT OF INTEREST STATEMENT

All authors have no conflict of interest with this work.

## Data Availability

The datasets are available from the corresponding author upon request.
